# When to Remove Adenoids

**Published:** 1901-11-09

**Authors:** 


### Hospital Clinics and Medical Progress.

### WHEN TO REMOVE ADENOIDS.

In a lecture recently delivered at the London Polyclinic, Sir Felix Semon [1] discussed the question of operating for adenoids. He drew attention to the curious vicissitudes which this operation had undergone. For the first ten years after its introduction by Wilhelm Meyer it remained practically unnoticed in this country. Then, after 1880, it gradually grew in popularity till, about 1890, the before despised operation had become the rage of the day. Ultimately it seemed as if every child required an operation and "when in doubt say adenoids" appeared to be a maxim of contemporaneous pediatrics. Soon after this, however, a new question began to be asked, namely, "Do not they come again?" and before long a wholesale condemnation began to be showered on the operation, largely, no doubt, in consequence of the frequency of the "recurrences" which arose from imperfect removal. Fashion is imperative; something new was wanted, and at last the revelation came in "breathing exercises." "I thank thee for that word" must have been the thought of many a professor of calisthenics, massage, gymnastics and corpulence curing, with a keen eye to business, who perceived that here a new and promising opening offered itself. "No sooner said than done; departments for breathing exercises were opened, the public flocked to them in their thousands, a roaring trade was and is being done at this moment, and children galore, who otherwise would have been operated upon by the doctors are now, if reports be true, cured or enormously benefited by so simple a method as breathing exercises. But are they?" In answer to this question Sir Felix Semon says:—"I do not believe for a moment that a single child which has got well marked adenoids has been, or ever will be, cured by breathing exercises, all reports to the contrary notwithstanding," and he adds that "the whole idea is preposterous." Not that he despises breathing exercises in their proper place, *i.e.*, when the obstruction has been removed or before it has become permanent. But that is quite another thing, and in such cases it is quite an old recommendation. Clearly the recent craze for breathing exercises and all they connote, is but a reaction against the tendency, which of late years has been so marked, to operate in all sorts of cases in which operation is quite unnecessary. In what cases, then, should we still advise operation, notwithstanding the present trend of fashion? Sir Felix Semon would subdivide adenoids into three classes, according as they cause (*a*) permanent, (*b*) periodical and transitory, or (*c*) no symptoms. The first class—the typical adenoids—embraces the cases in which respiratory obstruction, open mouth, snoring, thick voice, deafness, are all or some of them always present, and in which the altered type of respiration may have already led to the peculiar deformities of the face and chest, and to a generally debilitated state of health. In this class operation is absolutely indicated, and it is all the more strongly to be advised the further away the child is from the period of puberty, when sometimes spontaneous atrophy of the growth occurs. It was by its gratifying and sometimes astounding results in this class of case that the reputation of the operation was originally established. The second class (*b*) is the one in which there are sometimes real difficulties in coming to a correct conclusion. One has in such cases to consider which condition predominates. If a child be brought at its worst, but with a definite statement that these attacks only occur very rarely, that they last but a short time, and that in the intervals the child enjoys perfect health, and if, although there may be some soft swelling of the lymphoid tissue in the vault of the pharynx, there is no evidence of organic ear disease, operation may be postponed. But in proportion as the symptoms are more grave, as there is deafness, perforation of the tympana, otorrhœa, enlargement of the cervical glands, or a tuberculous family history, one would vote in favour of operation as a preventive of more serious events, even though the obstruction might not appear permanent. As to the third class (*c*), adenoids which do not cause symptoms do not require removal, and Sir Felix Semon specially raises his voice against the idea now so prevalent among so many specialists that various "reflex neuroses" depend upon, and are to be cured by the removal of, adenoids which are not in themselves producing any tangible respiratory or auditory symptoms. Finally, we are warned that any operation which it is decided to do for adenoids should be done thoroughly. Next to over-operating, nothing has so much damaged the reputation of the operation as the frequency with which so-called recurrences have been observed of recent years, and there is not the slightest doubt that what is commonly called a recurrence is, in the enormous majority of cases, the result of incomplete operation. It is the incredibly slipshod manner in which these operations are done that is the cause of the unsatisfactory results; a whiff of gas, a little scraping with the finger nail, and behold! the operation has been performed, and the parents are left in the belief that all has been done that could be done. Is it to be wondered at that recurrence so often takes place? As to the manner in which the operation should be done: Chloroform should be given, and the patient should be operated on in the recumbent position with the head well bent back over the end of the operation table. This method of arranging matters may be called "fussy" by some, but it is safe and yields infinitely fewer recurrences than that of the lightning operators. As to instruments, Sir Felix Semon almost always uses the curved and the straight Gottstein's curettes, and Hartmann's laterally-cutting curette, while in exceptional cases only does he employ Woakes's modification of Loewenberg's forceps.

[1] Brit. Med. Journ., Nov. 2.

